# The implementation and effectiveness of Integrated Psychological Therapy (IPT) in chronic middle-aged inpatients with schizophrenia

**DOI:** 10.1016/j.scog.2024.100330

**Published:** 2024-09-21

**Authors:** Aikaterini Poulou, Fotios Anagnostopoulos, Argiro Vatakis, Robert C. Mellon, Daniel R. Mueller

**Affiliations:** a Department of Psychology, Panteion University of Social and Political Sciences, Athens, Greece; b University Clinic of Psychiatry and Psychotherapy, University of Bern, Bern, Switzerland; c Department of Psychology, Faculty of Humanities, University of Fribourg, Fribourg, Switzerland

**Keywords:** Schizophrenia, Integrated Psychological Therapy (IPT), Cognitive impairments, Inpatients, Rehabilitation

## Abstract

**Introduction:**

Cognitive rehabilitation is essential for schizophrenia treatment since it improves function. Moreover, the relationship between cognitive rehabilitation and functioning is significantly affected by negative symptoms and social cognition. Integrated Psychological Therapy (IPT) is a promising approach that integrates interventions in neurocognition, social cognition, and functional level. This study examines IPT's efficacy in chronic middle-aged inpatients.

**Methods:**

A randomized controlled study involved 44 individuals with schizophrenia. Twenty-one IPT participants received 50 biweekly sessions and medication, while twenty-three control participants received treatment as usual/supportive therapy and pharmacotherapy. Pre- and post-intervention and six- and twelve-month follow-ups were arranged to assess neurocognition, social perception, psychopathology, and functioning using the Matrics Consensus Cognitive Battery, Social Perception Scale, Positive and Negative Syndrome Scale, and Global Assessment of Functioning.

**Results:**

Speed of processing, attention/vigilance, overall composite, and neurocognitive composite scores improved significantly in the IPT group. Social Perception Scale performance improved in all areas after the intervention and persisted for 6 months. Positive, negative, and total psychopathology symptoms decreased significantly post-intervention and at the 12-month follow-up, whereas participants' functioning improved significantly.

**Conclusions:**

Middle-aged chronic inpatients with schizophrenia may benefit from IPT in neurocognition, social perception, psychopathology, and functioning. This field of study may provide insight into schizophrenia treatment, hence further research is encouraged.

## Highlights

• An RCT was conducted on chronic middle-aged inpatients with schizophrenia in a private Greek psychiatric clinic.
• Neurocognition, Social Perception and Functioning improved significantly in the IPT group.
• Psychopathological symptoms decreased in the IPT group.
• IPT appears to be effective for middle-aged chronic inpatients with schizophrenia.
• The findings support implementing Integrated Psychological Therapy (IPT) in psychiatric settings.

## 1 Introduction

Schizophrenia is a mental illness characterized by cognitive, emotional, and behavioral impairments (American Psychological Association [APA], 2015; ICD-11, 2019). Schizophrenic symptomatology is usually divided into positive (i.e., any undesirable changes in behavior or thoughts, fixed or falsely held beliefs, and perception in the absence of any stimulus, such as delusions or hallucinations) and negative symptoms (i.e., lack of drive, social withdrawal, no interest in everyday social interactions, poverty of speech, emotional apathy and self-neglect; APA, 2015; ICD-11, 2019) with the latter having a strong connection with cognitive deficits (i.e., impairments of working memory, executive functions, verbal fluency, attention, and disturbances in the selection and processing of information; Balzan et al., 2014; Bustillo et al., 1995; Kontaxaki et al., 2014; Rosa et al., 2005; Rund, 1998). Additionally, questionnaire data demonstrate positive associations between cognitive dysfunction and negative symptom severity (Heydebrand et al., 2004; O'Leary et al., 2000). Like negative symptoms, cognitive deficits are associated with impaired effort-based decision making (EBDM; Cooper et al., 2019). Furthermore, Robison et al. (2020) posit that the co-occurrence of cognitive deficits and negative symptoms may contribute to the development of motivational impairments in individuals with schizophrenia. In their study, Saleh et al. (2023), showed that negative symptoms and cognitive impairments are linked to distinct motivational deficits particularly in individuals with treatment-resistant schizophrenia. Additionally, long-lasting functional deficits represent a challenge in the treatment of individuals with schizophrenia, given that 75–90 % of them suffer from cognitive impairments (Bell et al., 2013; Fett et al., 2013; Fioravanti et al., 2012; Green et al., 2012; Hovington et al., 2013; Roder and Mueller, 2008; Sachs, 2008; Ventura et al., 2013). Although atypical antipsychotics seem to be more effective than typical medication in improving cognitive function (Harvey et al., 2003; Harvey, 2008), further research is needed to understand how and to what extent these results can be achieved.

While schizophrenia is a globally debilitating mental health condition (Vos et al., 2020), it is crucial to prioritize not only symptom management but also functional improvement and social participation (Bighelli et al., 2023). Psychoeducation, acceptance and commitment therapy (ACT), cognitive behavioral therapy (CBT), and metacognitive training (MCT) may improve post-intervention positive symptoms, social functioning, and treatment compliance and reduced the risk of relapse and re-hospitalization, relative to control, according to Barnicot et al. (2020). A systematic review and meta-analysis of randomized controlled trials by Bighelli et al. (2023) examined how cognitive-behavioral therapy (CBT) for psychosis and third-wave CBT affected the functioning of people with schizophrenia. Their findings imply that both therapies can improve functioning compared to controls. Cognitive rehabilitation is important since it improves functional outcomes like job search. These associations are strongly mediated by negative symptoms and social cognition (Green, 1996; Green et al., 2012; Marder et al., 2003; Robertson et al., 2014; Schmidt et al., 2011; Strassnig et al., 2015; Ventura et al., 2014). While negative symptoms worsen the quality of life and functioning, individuals should receive skills-based training and integrated treatment approaches (Lutgens et al., 2017).

Mueller and Roder (2010) proposed a model incorporating patients' orientation to treatment as a combination of insight, knowledge, motivation, and self-efficacy. As a result, the Integrated Psychological Therapy (IPT) for schizophrenia was developed. IPT is a manualized group cognitive-behavioral therapy approach that combines interventions on neurocognition, social cognition, and social function (Roder et al., 2007, Roder et al., 2010). Its conceptualization assumes that cognitive deficits pervade higher levels of behavioral structure, such as social functioning. It comprises five subprograms (SP) with increasing levels of complexity, beginning with an intervention in neurocognition (SP1: Cognitive Differentiation: The ability to perceive several dimensions in a stimulus array; Bartunek et al., 1983) and social cognition (SP2: Social Perception: The ability to make accurate interpretations and inferences about other people from their general physical appearance, verbal, and nonverbal patterns of communication; Aronson et al., 2010), followed by an intervention in communication skills (SP3: Verbal Communication), social skills (SP4: Social Skills), and interpersonal problem-solving skills (SP5: Interpersonal Problem Solving; Roder et al., 2007). IPT appears to improve cognitive and social functioning in patients with schizophrenia, possibly reducing relapse rates (Alguero, 2006; Spaulding et al., 1999). Roder et al. (2006) demonstrated that IPT affected symptom-stabilized and post-acute patients. IPT patients with stable symptoms-maintained follow-up effects after therapy (Roder et al., 2006). This suggests that IPT is a promising treatment for schizophrenia.

Cohen et al. (2008) noted that positive symptoms decrease with age, negative symptoms and depression are similar in younger and older persons, and cognitive impairment increases with age. Through research, IPT has proved to be an effective therapeutic option for middle-aged patients (Mueller et al., 2013) as the goal-directed activities of the SPs appear to mobilize chronic patients by increasing their motivation to engage in treatment (Mueller et al., 2013; Roder et al., 2006, Roder et al., 2011). Mueller et al. (2013) found that middle-aged IPT individuals exhibited better cognitive therapeutic effects than younger ones. This inconsistency might have been due to the group setting of the IPT procedure (Mueller et al., 2013) and the fact that compensatory learning strategies (i.e., remembering verbal information by immediately repeating it for verification) seem to override the limited benefits of taking repeated cognitive tests in older people (>45 years old) (Granholm et al., 2010). Studies have demonstrated IPT's effectiveness in improving the neuropsychological and emotional states as well as the functioning of patients with schizophrenia (Aloi et al., 2020). Barlati et al. (2018) reported that IPT implementation maintained positive results in the aforementioned domains with a good cost-effectiveness ratio. In order to assess the benefits of IPT in middle-aged inpatients with chronic illness, aging, and cognitive deficits (Cohen et al., 2008; Mollon et al., 2020; Murman, 2015) empirical research is needed.

In this study, we evaluated the IPT group therapy program in middle-aged chronic inpatients. IPT (5 SPs) was anticipated to increase neurocognition, social cognition, and social functioning, and decrease negative symptoms. Therefore, it was expected that participants in the intervention group (IPT) would perform better than those in the control group (TAU) in neurocognition (i.e., speed of processing, attention/vigilance), social perception, psychopathology, and functioning. In addition, it was anticipated that these effects would endure at the six- and twelve-month follow-ups. A randomized controlled trial was designed and conducted to evaluate intervention impact, while reducing the risk of several potential biases, including selection, detection, and attrition bias. We were interested in quantifying the effect of assignment to the intervention at baseline, regardless of whether the intervention was received as intended (intention-to-treat analysis).

## 2 Materials and methods

### 2.1 Participants

The study was conducted in a private psychiatric clinic in Greece, where patients received psychiatric, psychological, and occupational therapy. The study was performed in accordance with the ethical standards laid down in the 2013 Declaration of Helsinki, it received ethical approval from the University's ethics committee 2/23–03–2021, while informed consent was obtained from all participants. The research participants were informed that they would not be compensated financially, that their involvement in the study was voluntary, and that they could withdraw from the study at any time without forfeiting their medical, pharmaceutical, psychotherapeutic, or occupational therapy services. Participants, along with their treating doctors or healthcare providers (where needed), willingly consented to participate in the study as long as it would not hinder their chances of being discharged before completion.

All participants met the following inclusion criteria: Diagnosis of schizophrenia according to DSM-V (APA, 2015) and ICD-11 (ICD-11, 2019), IQ between 80 and 110, assessed via WAIS-IV^GR^ (Wechsler Adult Intelligence Scale - 4th Edition, 2014; Wechsler, 2014), greater presence of negative symptomatology according to PANSS (PANSS: negative symptoms scores >28 and total symptoms scores >76; Kay et al., 1987; Lykouras et al., 2005), age range between 40 and 69 years at the beginning of the study, duration of the disease >5 years, no substance abuse, no organic brain disease, no relapse of positive symptoms one month before the study admission, according to their medical records and doctors' reports. Changes in medications were allowed before the study intake, where necessary, but not while the IPT was implemented.

**Fig. 1:**
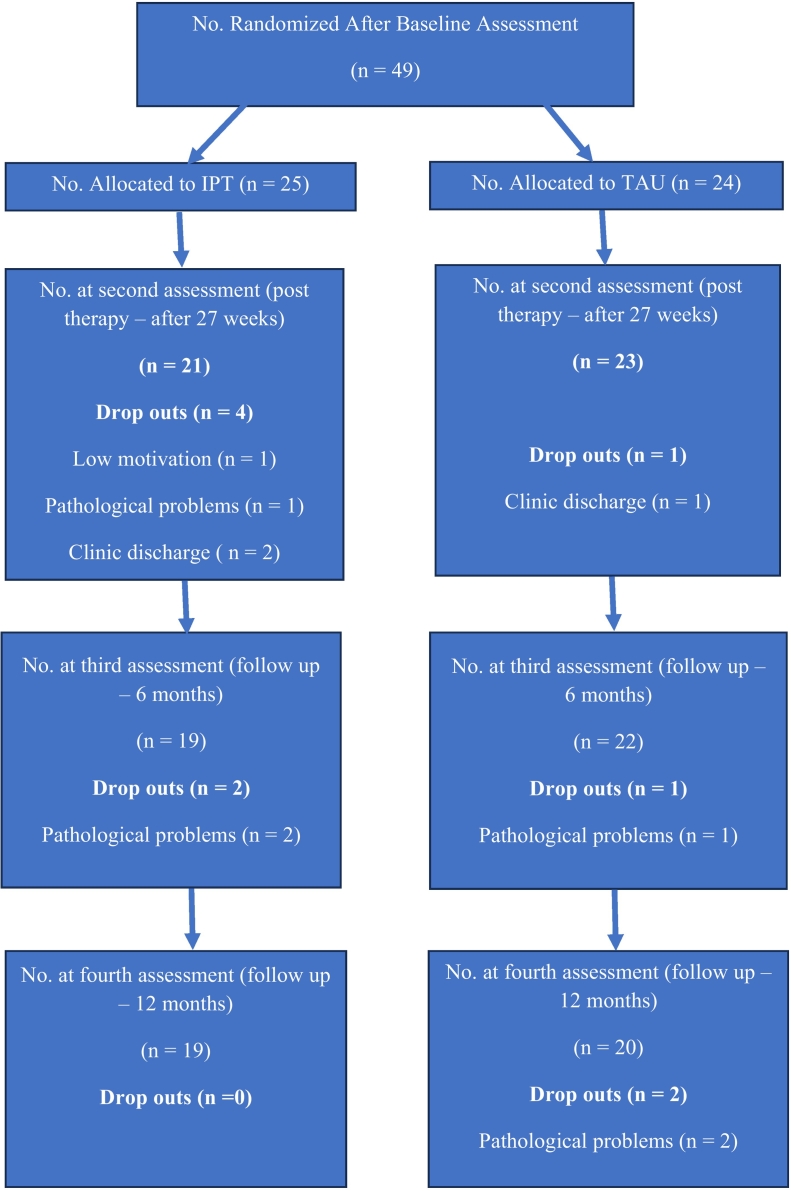
Consort diagram: Flow diagram of subject progress through phases of the randomized controlled trial for the Integrated Psychological Therapy (IPT) and Treatment As Usual (TAU) group.

From a clinic population of 120 patients, 49 middle-aged chronic inpatients diagnosed with schizophrenia satisfied the research criteria and were eligible to participate. The sample consisted of 20 females and 29 males, with mean age of 60.14 years old (SD = 5.96, age range from 44 to 68 years old), mean years of illness 32.53 years (SD = 8.09, illness range from 11 to 45 years), mean years of hospitalization 7.61 years (SD = 4.58, hospitalization range from 1 to 20 years) and mean IQ score 86.59 (SD = 7.83, range from 80 to 107). The intervention group comprised 25 patients, whereas the control group comprised 24 patients. Four patients from the IPT group and one patient from the TAU group withdrew before the post-intervention evaluation and were therefore excluded from further analysis. At the 6-month follow-up, one participant from the IPT group and one participant from the TAU group withdrew. At the 12-month follow-up, two individuals from the TAU group withdrew. The reasons for dropping out were low motivation: attendance rate < 50 % or the absence of 4 continuing sessions (post intervention assessment: IPT *n* = 1, TAU *n* = 0), pathological problems (post intervention assessment: IPT n = 1, TAU n = 0; 6-month follow up: IPT *n* = 2, TAU n = 1; 12-month follow up TAU n = 2), and clinic discharge (post intervention: IPT n = 2, TAU n = 1; see Fig. 1).

### 2.2 Study design

In this randomized controlled trial (RCT), an independent rater used the Statistical Package for the Social Sciences (SPSS) for a randomization procedure after the baseline (T1) assessment. Twenty-five patients were allocated to IPT and twenty-four to TAU group. Three subgroups of IPT and TAU individuals were formed. The IPT group had two 8-person subgroups and one 9-person subgroup, while the TAU group included three 8-person subgroups. The 27-week intervention was followed by a second evaluation (T2). The third assessment (T3) was conducted 6 months following intervention, and the fourth assessment (T4) was scheduled 12 months later. Two blinded MD and MSc Psychology raters performed the psychological assessments.

### 2.3 Intervention

Fifty hourly bi-weekly IPT sessions were held for intervention group members. All 5 SPs of IPT were employed (i.e., Cognitive Differentiation, Social Perception, Verbal Communication, Social Skills, and Interpersonal Problem Solving). The first session covered psychoeducation with Zubin and Spring's stress–vulnerability model (Zubin and Spring, 1977), suggesting that each person is endowed with a degree of vulnerability (e.g. genetic inheritance, acquired propensities), that under certain circumstances, can lead to a schizophrenic episode. In the last session, IPT members provided program feedback. The TAU group attended 50 biweekly supportive therapy sessions to discuss social concerns and receive individual counseling. In addition to treatment, case management helped participants reconnect with reality and cope with their daily lives at the clinic. Both groups received standard and/or atypical antipsychotics.

An MSc psychologist, trained in Cognitive Behavioral Therapy (CBT) and IPT procedure, implemented the intervention in the IPT and control groups. The intervention group was supervised by Dr. Rakitzi (PhD, Dipl. Psychologist, IPT instructor) to ensure compliance with the Greek IPT manual (Roder et al., 2007).

The final dataset included 44 records, with a total of 277 missing values (3.8 %). Analysis with Little's MCAR test indicated that values were missing completely at random (χ^2^(34) = 29.04, *p* = .710; there was no systematic pattern of missingness that would imply a clinical reason or dependency between variables having missing values; Little, 1988). Multiple imputation (MI; a statistical imputation method that is effective when data are missing completely at random; Jacobsen et al., 2017) and the predictive mean matching method (PMM; Hong and Lynn, 2020) were employed using IBM SPSS v.28 to impute missing values, creating five imputed datasets. The five imputed datasets were analyzed separately, and combined results (pooled results) were presented. The effect size was calculated with partial eta squared' SPSS index (small, η^2^ = 0.01; medium, η^2^ = 0.06; large, η^2^ = 0.14).

### 2.4 Measures

#### 2.4.1 Neurocognition

The neurocognitive domain was evaluated using the Matrics Consensus Cognitive Battery (MCCB), which measures cognitive domains associated with schizophrenia and related disorders (Green et al., 2008; Kern et al., 2008; Nuechterlein et al., 2008). The MCCB is a standardized battery including 10 individual tests that assess cognitive performance across seven domains: speed of processing, attention/vigilance, working memory, verbal learning, visual learning, social cognition and reasoning and problem-solving. The test–retest reliability of the MCCB composite score was ICC = 0.88 (Keefe et al., 2011).

The cognitive domain of speed of processing was measured by three sub-tests: Brief Assessment of Cognition in Schizophrenia (BACS): Symbol Coding (Keefe et al., 2004), Category Fluency: Animal Naming (Fluency; Lezak, 1995), and Trail Making Test (TMT): Part A (Strauss et al., 2006). The attention/vigilance was measured by Continuous Performance Test – Identical Pairs (CPT-IP), MATRICS International Version 2 (Cornblatt et al., 1988; Nuechterlein and Green, 2006), while the domain of working memory was evaluated by the Wechsler Memory Scale -Third Edition (WMS – III): Spatial Span (Wechsler, 1997) and Letter Number Span (LNS; Baddeley et al., 1986; Gold et al., 1997; Nuechterlein and Green, 2006). Verbal learning was evaluated using the Hopkins Verbal Learning Test – Revised (HVLT – R; Brandt and Benedict, 2001), visual learning was measured by the Brief Visuospatial Memory Test – Revised (BVMT – R; Benedict, 1997), and the domain of reasoning and problem-solving was evaluated using the Neuropsychological Assessment Battery (NAB): Mazes (Stern and White, 2003). Lastly, the domain of social cognition was evaluated by the Mayer – Salovey – Caruso Emotional Intelligence Test (MSCEIT): Managing Emotions (D & H; Mayer et al., 2002; Nuechterlein et al., 2008; Kern et al., 2008; Green et al., 2008).

The English MCCB battery was translated into Greek for non-commercial use. The translation procedure included a forward translation to the target language, a reverse translation to English, and discrepancy resolution. After the preceding steps, MATRICS Assessment, Inc. and the intellectual property owners allowed us to use the MCCB translation in our study.

#### 2.4.2 Social cognition

The Greek version of the Social Perception Scale (SPS; García et al., 2003; Rakitzi, 2007; Ruiz et al., 2005) assessed the three main aspects of the IPT's Social Perception Program: stimuli identification, image interpretation, and title assignment.

#### 2.4.3 Psychopathology

Positive, negative, and general (psychopathological) symptoms were assessed using the Greek version of the Positive and Negative Syndrome Scale (PANSS; Kay et al., 1987; Lykouras et al., 2005). Reliability coefficients ranged from 0.82 to 1.00 for the positive scale, 0.56 to 0.86 for the negative scale, and 0.71 to 1.00 for the general psychopathology scale (Kay et al., 1987; Lykouras et al., 2005). An experienced, blinded rater with an MD degree conducted the PANSS interview.

#### 2.4.4 Functional outcome

Psychosocial functioning was assessed using the Global Assessment of Functioning (GAF; APA, 2004; Endicott et al., 1976; Madianos, 1987). The internal consistency reliability coefficient for the scale was equal to 0.74, with SE = 5.8 (*p* < .001). The blinded rater with an MD degree scored the GAF scale.

## 3 Results

This study included 44 individuals (23 TAU and 21 IPT), with baseline characteristics shown in Table 1. There were no significant differences between the two groups. GAF score was only slightly significant (*p* = .046), with the IPT group scoring higher (M = 30.38, SD = 7.68) than the TAU group (M = 26.35, SD = 5.73).

**Table 1:** Patients characteristics at baseline (*N* = 44).

	TAU (*n* = 23)	IPT (*n* = 21)	*p*
Gender (% male, *n*)	52.2 % (12)	57.1 % (12)	.741^a^
Age at baseline (years), *M*(*SD*)	60.39 (6.01)	61.58 (5.03)	.571^b^
Duration of illness at baseline (years), *M*(*SD*)	32.22 (8.81)	33.71 (7.7)	.604^b^
Duration of Hospitalization at baseline (years), *M*(*SD*)	6.61 (4.7)	8.62 (3.72)	.096^b^
Years of Education, *M*(*SD*)	11.04 (3.34)	12.14 (3.26)	.229^b^
IQ score, *M*(*SD*)	83.87 (5.75)	87.33 (7.09)	.061^b^
GAF, *M*(*SD*)	26.35 (5.73)	30.38 (7.68)	.046^b^
PANSS Total Score, *M*(*SD*)	111.09 (8.97)	106.57 (12.01)	.118^b^
Only typical medication (%)	39.1 % (9)	28.6 % (6)	.460^a^
Only non-typical medication (%)	13.0 % (3)	14.3 % (3)	.905^a^
Both typical and non-typical medication (%)	47.8 % (11)	57.1 % (12)	.537^a^
Medication (chlorpromazine equivalent dose), *M*(*SD*)	113.04 (157.55)	161.9 (162.72)	.254^b^
Medication (clozapine equivalent dose), *M*(*SD*)	60.87 (147.68)	52.38 (163.15)	.768^b^
Haloperidol equivalent dose, *M*(*SD*)	28.04 (19.35)	28(25.05)	.941^b^

Note: *M*: mean value, *SD*: standard deviation.

a *p*-Value of chi-square test.

b *p*-Value of Mann-Whitney tests.

The pooled mean and standard error values of neurocognition (MCCB), social perception (SPS), symptoms (PANSS) and functionality (GAF) variables per assessment (T1-T4) are presented in Table 2, Table 3 for the TAU (*n* = 23) and IPT (*n* = 21) groups respectively. Pooled effect size and power values were estimated as the median value produced from Repeated Measures ANOVA with Bonferroni correction (Field, 2013) by the five MI completed datasets.

**Table 2:**
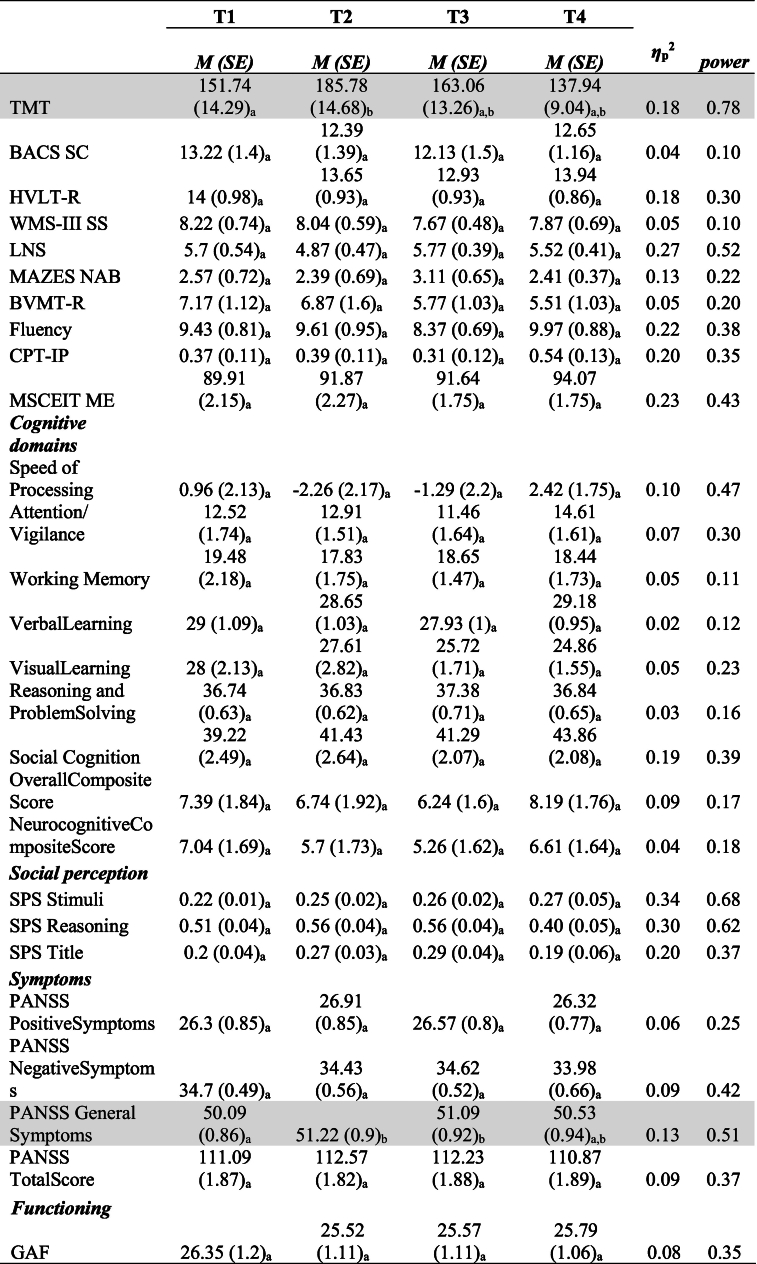
Pooled Repeated Measures ANOVA results along with Bonferroni Multiple Comparisons for neurocognition (MCCB), social perception (SPS), symptoms (PANSS) and functionality (GAF) of TAU group (*n* = 23).

Note. *M*(*SE*): Pooled mean and standard error values from 5 iterations of PMM imputation. T1, T2, T3, T4: assessment points pre therapy, post therapy, at 6 months follow-up and at 12 months follow-up. Values in the same row not sharing the same subscript are significantly different at *p* < .05. Shaded lines indicate variables with statistically significant pooled differences between assessments as indicated by pairwise comparisons using the Bonferroni correction. Effect sizes and power are estimated as the median of 5 iterations.

**Table 3:**
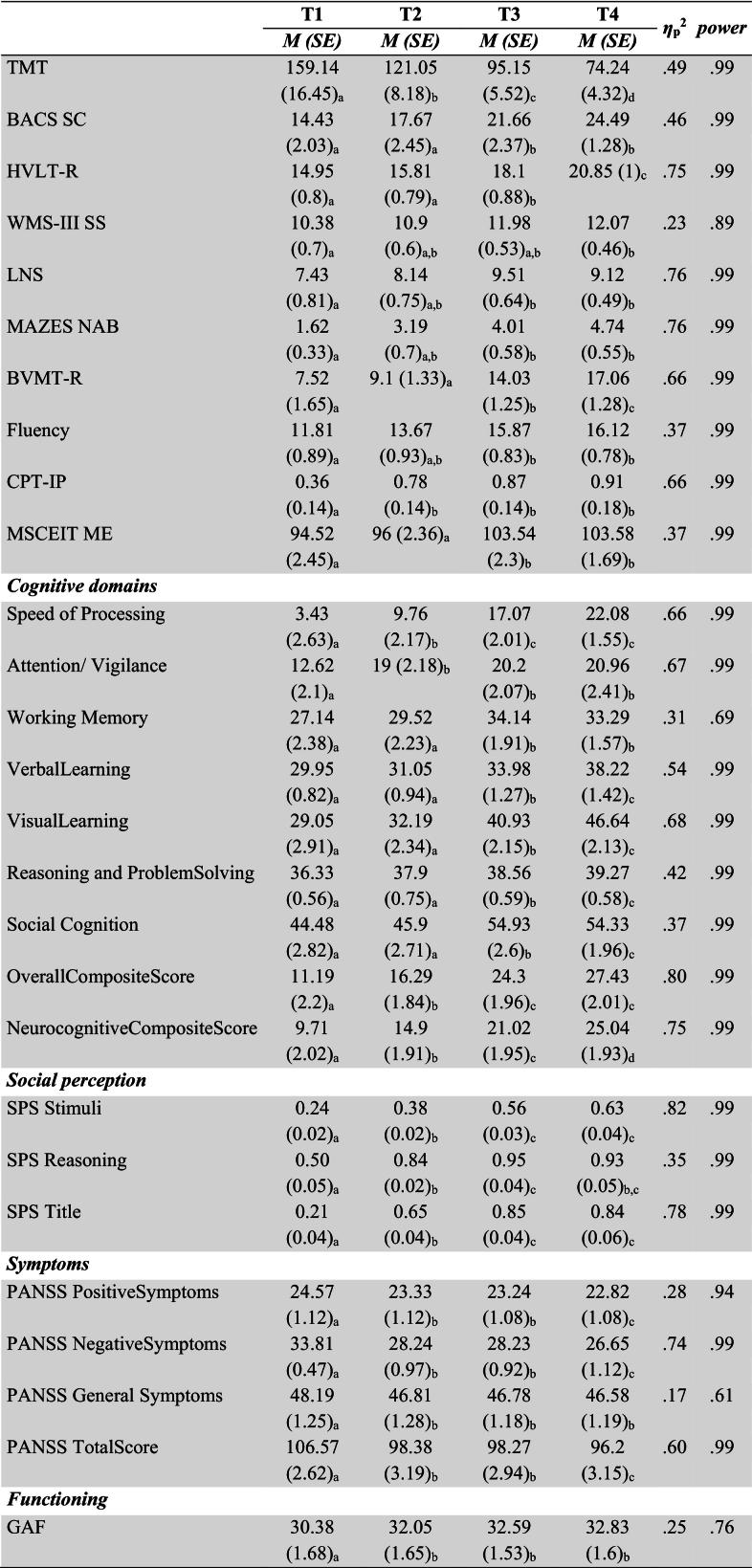
Repeated Measures ANOVA along with Bonferroni Multiple Comparisons for neurocognition (MCCB), social perception (SPS), symptoms (PANSS) and functionality (GAF) of IPT group (*n* = 21).

Note. *M*(*SE*): Pooled mean and standard error values from 5 iterations of PMM imputation. T1, T2, T3, T4: assessment points pre therapy, post therapy, at 6 months follow-up and at 12 months follow-up. Values in the same row not sharing the same subscript are significantly different at p < .05. Shaded lines indicate variables with statistically significant pooled differences between assessments as indicated by pairwise comparisons using the Bonferroni correction. Effect sizes and power are estimated as the median of 5 iterations.

As shown in Table 2, the measures of TAU group remained the same throughout treatment, except for an increase that was detected post treatment in TMT (longer completion time, T2; M = 185.78, SE = 14.68, *p* < .05) and PANSS General Symptoms (rise in general psychopathology, T2; M = 51.22, SE = 0.9, p < .05).

On the other hand, significant changes were detected in the IPT group with strong effect sizes, as presented in Table 3. There is a noticeable enhancement in the scores of participants across all sub-scales during the measurements. The intervention (at T2) resulted in a significant improvement in participants' cognitive performance across multiple domains, including TMT (Μ = 121.5, SE = 8.18, p < .05, *η*^2^ = 0.49), CPT – IP (Μ = 0.78, SE = 0.14, p < .05, *η*^*2*^ = 0.66), speed of processing (Μ = 9.76, SE = 2.17, p < .05, *η*^2^ = 0.66), attention/vigilance (M = 19.00, SE = 2.18, p < .05, *η*^2^ = 0.67), overall composite score (M = 16.29, SE = 1.84, p < .05, *η*^2^ = 0.80), and neurocognitive composite score (M = 14.9, SE = 1.91, p < .05, *η*^2^ = 0.75). SPS Stimuli, Reasoning and Title Scores increased significantly post therapy and again at 6 months follow-up (partial *η*^2^ = 0.82, 0.35 and 0.78 respectively). PANSS Positive and Negative Symptoms Score as well as the PANSS Total Score decreased significantly post therapy and again at 12 months follow-up compared to pre therapy assessment (partial *η*^*2*^ = 0.28, 0.74 and 0.60 respectively), while PANSS General Symptoms Score decreased significantly post therapy (partial *η*^*2*^ = 0.17). Finally, participants' functional outcomes improved significantly post therapy through GAF (M = 32.05, SE = 1.65, p < .05, *η*^*2*^ = 0.25) compared to pre therapy assessment.

The results of Table 4 for the Linear Mixed Model (LMM) analysis (Magezi, 2015), showed that at T4 there was a significant decrease in the TMT of IPT group (they were faster) compared to T1(mean difference of 66.9 s; SE = 24.6 s). Moreover, the BACS SC score of IPT group in T4 was higher by 11.4 points (SE = 2.88) compared to T1 and by 7.4 points (SE = 2.95) compared to T2. HVLT -R at T4 was higher for the IPT group by 4.77 points (SE = 1.7) compared to T2 and MAZES NAB at T4 was higher by 3.0 points (SE = 1) compared to T1. BVMT – R at T4 was higher by 11.5 points (SE = 2.2) compared to T1 and by 9.7 points (SE = 2.25) compared to T2 while Fluency at T4 was higher by 3.7 points (SE = 1.63) compared to T1. Moreover, female participants were slower on TMT by 29 s (SE = 8.9 s) and they had a lower MAZES NAB score by 1.5 points (SE = 0.6) compared to male participants. IQ score and years of education were positively associated with an increase in BACS SC, BVMT – R, LNS and Fluency scores, while IQ score was also positively associated with an increase in HVLT – R, WMS – III and CPT – IP scores. On the other hand, haloperidol consumption was associated with a decrease in HVLT – R (b = −2.16, p < .05) and CPT – IP (b = −0.25, *p* < .01) scores. Clozapine was associated with a decrease in WMS – III, LNS and MSCEIT ME while Chlorpromazine consumption was related to a decrease in WMS – III scores.

**Table 4:** Pooled Results of Linear Mixed Models for test measurements.

	TMT	BACS SC	HVLT-R	WMS-III SS	LNS	MAZES NAB	BVMT-R	Fluency	CPT-IP	MSCEIT ME
Intercept, Estimate (SE)	−64.52 (61.92)	8.11 (7.93)	−2.78 (5.01)	−0.41 (3.1)	−0.94 (3.17)	−3.75 (3.68)	3.03 (6.15)	0.19 (5.18)	0.4 (0.7)	−77.59 (11.88)⁎⁎⁎
Group, Estimate (SE)	61.72 (14.93)⁎⁎⁎	−11.57 (2.15)⁎⁎⁎	−5.95 (1.38)⁎⁎⁎	−3.7 (0.98)⁎⁎⁎	−3.07 (0.72)⁎⁎⁎	−2.21 (0.73)⁎⁎	−10.98 (1.39)⁎⁎⁎	−5.75 (1.25)⁎⁎⁎	−0.26 (0.18)	−5.95 (2.75)⁎
T1 vs T4, Estimate (SE)	59.65 (23.8)⁎⁎	−9.47 (3.1)⁎⁎	−4.99 (1.63)⁎⁎	−1.47 (1.15)	−0.87 (1.01)	−2.92 (1.16)⁎	−9.02 (2.46)⁎⁎⁎	−2.91 (1.63)	−0.29 (0.23)	−5.28 (3.95)
T2 vs T4, Estimate (SE)	30.61 (18.12)	−6.26 (2.81)⁎	−4 (1.48)⁎⁎	−1.02 (0.98)	−0.58 (0.82)	−1.63 (1.06)	−7.46 (2.16)⁎⁎⁎	−0.6 (1.53)	0.04 (0.21)	−4.91 (3.64)
T3 vs T4, Estimate (SE)	16.65 (17.54)	−3.14 (2.44)	−2.72 (1.39)	0.35 (0.84)	0.21 (0.7)	−0.4 (0.81)	−2.9 (1.41)⁎⁎⁎	0.08 (1.13)	−0.01 (0.17)	0.61 (2.73)
Group*T1 vs T4, Estimate (SE)	66.9 (24.56)⁎	−11.42 (2.88)⁎⁎⁎	−6 (1.68)	1.77 (1.21)	1.72 (1.04)	−3.04 (1.07)⁎⁎	−11.54 (2.23)⁎⁎⁎	−3.69 (1.63)⁎	−0.35 (0.23)	−3.85 (3.9)
Group*T2 vs T4, Estimate (SE)	3.42 (20.96)	−7.43 (2.95)⁎⁎	−4.77 (1.7)⁎⁎	−1.11 (1.15)	0.24 (0.96)	−1.34 (1.14)	−9.68 (2.25)⁎⁎⁎	−1.94 (1.78)	−0.03 (0.24)	4.44 (4.18)
Group*T3 vs T4, Estimate (SE)	8.61 (21.03)	−3 (3.22)	−2.06 (1.77)	−0.45 (1.15)	0.04 (1.02)	−1.01 (1.17)	−3.47 (1.94)	−1.63 (1.65)	−0.19 (0.24)	−2.25 (3.76)
Female vs Male, Estimate (SE)	29.28 (8.86)⁎⁎⁎	−0.8 (1.1)	1.84 (0.65)⁎⁎	−0.63 (0.44)	0.45 (0.42)	−1.49 (0.57)⁎	−0.76 (0.85)	−1.05 (0.63)	−0.01 (0.1)	2.09 (1.58)
Education years, Estimate (SE)	−1.37 (1.34)	0.54 (0.18)⁎⁎	0.1 (0.1)	−0.02 (0.07)	0.22 (0.06)⁎⁎⁎	0.05 (0.07)	0.36 (0.14)⁎	−0.24 (0.1)⁎	0.01 (0.01)	0.27 (0.25)
IQ score, Estimate (SE)	−0.38 (0.69)	0.46 (0.09)⁎⁎⁎	0.22 (0.06)⁎⁎⁎	0.19 (0.03)⁎⁎⁎	0.11 (0.04)⁎⁎	0.05 (0.04)	0.28 (0.07)⁎⁎⁎	0.21 (0.06)⁎⁎⁎	0.02 (0.01)⁎⁎	0.24 (0.13)
Combined medication, Estimate (SE)	17.06 (20.72)	−0.22 (1.96)	0.26 (1.12)	−0.18 (0.8)	−0.73 (0.8)	−0.51 (0.85)	−0.04 (1.85)	0.76 (1.12)	−0.15 (0.16)	−1.73 (3.02)
Haloperidol, Estimate (SE)	13.93 (11.68)	−2.26 (1.5)	−2.16 (0.88)⁎	−0.27 (0.62)	−0.16 (0.58)	0.54 (0.68)	−0.97 (1.26)	0.47 (0.87)	−0.25 (0.12)⁎	−3.1 (2.17)
Chlorpromazine, Estimate (SE)	−9.28 (9.92)	0.11 (1.24)	−0.51 (0.68)	−0.96 (0.45)⁎	−0.57 (0.39)	−0.2 (0.49)	−1.61 (0.92)	−0.96 (0.65)	−0.12 (0.1)	0.21 (1.69)
Clozapine, Estimate (SE)	8.58 (12.21)	−2.04 (1.61)	−1.03 (0.92)	−1.57 (0.64)⁎	−1.61 (0.57)⁎⁎	−0.54 (0.66)	−0.42 (1.27)	−0.53 (0.9)	−0.11 (0.13)	−6.5 (2.28)⁎⁎
Years of illness, Estimate (SE)	0.61 (20.52)	−0.17 (2.59)	−0.05 (1.48)	−0.04 (0.97)	−0.08 (0.86)	−0.05 (1.06)	−0.16 (2.05)	0.01 (1.46)	−0.02 (0.21)	−0.06 (3.71)
Years of hospitalization, Estimate (SE)	2.51 (20.53)	−0.43 (2.59)	0.04 (1.48)	−0.08 (0.97)	−0.03 (0.86)	−0.09 (1.06)	−0.02 (2.05)	−0.04 (1.46)	0.01 (0.21)	0.44 (3.71)
Calls, Estimate (SE)	3.21 (22.17)	−1.12 (2.79)	−0.87 (1.58)	−0.7 (1.04)	−1.28 (0.93)	−1.32 (1.18)	−1.83 (2.2)	−0.26 (1.55)	−0.09 (0.23)	−3.81 (4.01)
Visits, Estimate (SE)	−13.96 (23.49)	0.91 (3.02)	1.03 (1.71)	−0.33 (1.19)	0.39 (0.98)	−0.25 (1.27)	0.53 (2.49)	1.89 (1.68)	0.17 (0.24)	1.95 (4.19)
Diet, Estimate (SE)	21.11 (21.02)	−3.56 (2.65)	0.26 (1.56)	−1.03 (1)	0.18 (0.88)	−0.1 (1.09)	−0.64 (2.11)	−0.42 (1.49)	0.12 (0.22)	3.56 (3.79)
Residual of random effects, Variance (SE)	0.000 (3.93)	0.000 (0.518)	0.000 (0.299)	0.000 (0.167)	0.000 (0.079)	0.000 (0.143)	0.000 (0.230)	0.000 (0.205)	0.000 (0.043)	0.056 (0.314)

Note. Random effects of Years of illness, Years of hospitalization, Calls, Visits, Diet were included in the model.

⁎⁎⁎ *p* < .001.

⁎⁎ *p* < .01.

⁎ *p* < .05.

The results of Table 5 for LMM analysis, showed that at T4 there was a significant increase in the Speed of Processing of the IPT group compared to T1 (mean difference of 16.9 points; SE = 3.96) and to T2 (mean difference of 7.53 points; SE = 3.8). Attention/Vigilance at T4 was higher by 5.25 points (SE = 3.4) compared to T1, Working Memory was higher by 6.96 points (SE = 3.25) compared to T1, Verbal Learning was higher by 7.91 points (SE = 2.0) compared to T1 and by 6.44 points (SE = 2.0) compared to T2. Continuing, Visual Learning at T4 was higher by 20.13 points (SE = 3.9) compared to T1, Overall Composite score at T4 was higher by 14.9 points (SE = 3.1) compared to T1 and by 9.3 points (SE = 3.1) compared to T2, while Neurocognitive Composite score at T4 was higher by 15.1 points (SE = 2.9) compared to T1 and by 8.7 points (SE = 3.0) compared to T2.

**Table 5:** Pooled Results of Linear Mixed Models for Cognitive Domains.

	Speed of Processing	Attention/Vigilance	Working Memory	Verbal Learning	Visual Learning	Reasoning and Problem Solving	Social Cognition	Overall Composite Score	Neurocognitive Composite Score
Intercept, Estimate (SE)	−16.72 (10.16)	−1.41 (10.21)	−8.31 (8.61)	14.03 (5.63)⁎	0.63 (10.98)	28.7 (3.68)⁎⁎⁎	28.74 (13.84)⁎	−20.22 (8.38)⁎	−22.2 (8.04)⁎⁎
Group, Estimate (SE)	−18.32 (2.97)⁎⁎⁎	−4.12 (2.65)	−13.61 (2.48)⁎⁎⁎	−7.48 (1.66)⁎⁎⁎	−19.13 (2.46)⁎⁎⁎	−2.28 (0.87)⁎	−6.81 (3.2)⁎	−16.53 (2.38)⁎⁎⁎	−16.12 (2.39)⁎⁎⁎
T1 vs T4, Estimate (SE)	−13.63 (3.76)⁎⁎⁎	−4.2 (3.4)	−4.19 (3.12)	−6.65 (1.95)⁎⁎⁎	−15.87 (4.3)⁎⁎⁎	−2.38 (1.11)⁎	−6.17 (4.6)	−12.19 (3.08)⁎⁎⁎	−11.42 (2.85)⁎⁎⁎
T2 vs T4, Estimate (SE)	−7.82 (3.19)⁎	0.93 (3.11)	−2.42 (2.63)	−5.33 (1.79)⁎⁎	−12.49 (3.75)⁎⁎	−0.83 (1.04)	−6.06 (4.22)	−7.81 (2.77)⁎⁎	−6.75 (2.6)⁎
T3 vs T4, Estimate (SE)	−4.47 (2.88)	−0.39 (2.52)	0.64 (2.28)	−3.81 (1.7)⁎	−5.28 (2.47)⁎	−0.79 (0.86)	0.9 (3.19)	−3.03 (2.17)	−3.52 (2.14)
Group*T1 vs T4, Estimate (SE)	16.9 (3.96)⁎⁎⁎	5.25 (3.43)⁎⁎	6.96 (3.25)⁎	7.91 (2)⁎⁎⁎	20.13 (3.97)⁎⁎⁎	2.82 (1.08)⁎⁎	4.37 (4.56)	14.91 (3.05)⁎⁎⁎	15.09 (2.9)⁎⁎⁎
Group*T2 vs T4, Estimate (SE)	7.53 (3.8)⁎	−0.56 (3.54)	3.07 (3.09)	6.44 (2.04)⁎⁎	16.62 (4)⁎⁎⁎	1.37 (1.16)	5.3 (4.86)	9.31 (3.09)⁎⁎	8.69 (2.98)⁎⁎
Group*T3 vs T4, Estimate (SE)	0.93 (4.01)	−2.69 (3.51)	−0.12 (3.19)	2.94 (2.24)	5.96 (3.4)	1.38 (1.17)	−2.7 (4.4)	1.43 (3.03)	2.25 (3.03)
Female vs Male, Estimate (SE)	−4.6 (1.41)⁎⁎	1.76 (1.39)	1.82 (1.25)	0.52 (0.78)	−1.19 (1.51)	1.82 (0.46)⁎⁎	1.24 (1.85)	0.35 (1.16)	−0.06 (1.11)
Education years, Estimate (SE)	0.13 (0.22)	0.16 (0.21)	0.29 (0.19)	0.08 (0.12)	0.51 (0.25)⁎	−0.07 (0.07)	0.34 (0.29)	0.36 (0.18)	0.27 (0.17)
IQ score, Estimate (SE)	0.54 (0.12)⁎⁎⁎	0.32 (0.12)⁎⁎	0.59 (0.1)⁎⁎⁎	0.29 (0.06)⁎⁎⁎	0.59 (0.12)⁎⁎⁎	0.11 (0.04)⁎⁎	0.25 (0.16)	0.62 (0.09)⁎⁎⁎	0.63 (0.09)⁎⁎⁎
Combined medication, Estimate (SE)	−1.18 (2.81)	−2.11 (2.39)	−1.16 (2.32)	0.4 (1.27)	0.31 (3.3)	−0.09 (0.76)	−2.06 (3.53)	−1.31 (2.1)	−1.02 (1.88)
Haloperidol, Estimate (SE)	−3.42 (1.89)	−3.85 (1.78)⁎	−0.99 (1.8)	−3 (1.04)⁎⁎	−2.98 (2.31)	−0.66 (0.62)	−3.41 (2.49)	−4.11 (1.63)⁎	−3.43 (1.54)⁎
Chlorpromazine, Estimate (SE)	−0.21 (1.59)	−1.9 (1.4)	−3.05 (1.31)⁎	−0.89 (0.8)	−3.34 (1.65)	−0.45 (0.46)	0.25 (1.94)	−2.56 (1.22)⁎	−2.98 (1.16)⁎
Clozapine, Estimate (SE)	−3.6 (2.06)	−2.28 (1.95)	−7.26 (1.86)⁎⁎⁎	−0.97 (1.1)	−2.41 (2.26)	−2.37 (0.64)	−6.85 (2.66)⁎	−6.14 (1.68)⁎	−4.75 (1.6)⁎
Years of illness, Estimate (SE)	−0.04 (3.26)	−0.25 (3.13)	−0.17 (2.74)	−0.05 (1.8)	−0.15 (3.64)	0.08 (1.03)	−0.11 (4.32)	−0.16 (2.67)	−0.14 (2.55)
Years of hospitalization, Estimate (SE)	−0.49 (3.26)	0.11 (3.13)	−0.25 (2.74)	0.06 (1.8)	−0.04 (3.64)	−0.09 (1.03)	0.52 (4.32)	−0.04 (2.67)	−0.18 (2.55)
Calls, Estimate (SE)	−0.47 (3.56)	−1.15 (3.36)	−3.22 (2.93)	−0.65 (1.91)	−2.43 (3.9)	−0.17 (1.11)	−4.51 (4.67)	−3.03 (2.86)	−1.88 (2.71)
Visits, Estimate (SE)	4.54 (3.9)	2.94 (3.56)	0.9 (3.19)	1.77 (2.08)	1.67 (4.34)	0.57 (1.19)	1.84 (4.87)	3.27 (3.22)	3.18 (3.05)
Diet, Estimate (SE)	−5.14 (3.33)	1.49 (3.21)	−2.33 (2.82)	−0.26 (1.88)	−2.47 (3.73)	−1.16 (1.07)	4.5 (4.41)	−1.37 (2.75)	−2.52 (2.61)
Residual of random effects, Variance (SE)	0.000 (0.401)	0.000 (0.639)	0.000 (0.336)	0.000 (0.471)	0.000 (0.496)	0.000 (0.151)	0.000 (0.370)	0.000 (0.401)	0.000 (0.466)

Note. Random effects of Years of illness, Years of hospitalization, Calls, Visits, Diet were included in the model.

⁎⁎⁎ *p* < .001.

⁎⁎ *p* < .01.

⁎ *p* < .05.

Female participants had on average a lower speed of processing by 4.6 points (SE = 1.41) compared to male ones, but on the other hand, they had a higher score on reasoning and problem solving compared to male participants by 1.8 points (SE = 0.5).

Haloperidol consumption was related to a decrease in Attention/Vigilance by 3.85 points (SE = 1.78), in Verbal learning by 3.0 points (SE = 1.04), in Overall Composite score by 4.1 points (SE = 1.63) and in Neurocognitive Composite score by 3.4 points (SE = 1.54). Moreover, Chlorpromazine and Clozapine were related to a decrease in Working Memory by 3.05 (SE = 1.31) and 7.26 (SE = 1.86) points respectively, in Overall Composite score by 2.6 (SE = 1.22) and 6.1 (SE = 1.68) points, and in Neurocognitive Composite score by 2.98 (SE = 1.16) and 4.8(SE = 1.6) points respectively.

The pooled results of LMM for social perception, symptoms and functioning are presented in Table 6. SPS Stimuli at T4 was higher by 0.33 points (SE = 0.04) compared to T1 and by 0.22 points (SE = 0.04) compared to T2. SPS Reasoning at T4 was higher by 0.5 points (SE = 0.09) compared to T1 and by 0.25 points (SE = 0.08) compared to T2, while SPS Title at T4 was higher by 0.6 points (SE = 0.09) compared to T1 and by 0.28 points (SE = 0.09) compared to T2.

**Table 6:** Pooled Results of Linear Mixed Models for Social perception, Symptoms and Functioning.

	SPS Stimuli	SPS Reasoning	SPS Title	PANSS Positive Symptoms	PANSS Negative Symptoms	PANSS General Symptoms	PANSS Total Score	GAF
Intercept, Estimate (SE)	−0.2 (0.13)	0.63 (0.24)⁎	−0.43 (0.28)	15.94 (4.73)⁎⁎⁎	33.3 (3.25)⁎⁎	53.95 (4.73)⁎⁎	103.63 (10.5)⁎⁎	22.84 (6.72)⁎⁎⁎
Group, Estimate (SE)	0.34 (0.04)⁎⁎⁎	0.52 (0.06)⁎⁎⁎	0.64 (0.08)⁎⁎⁎	−3.46 (1.21)⁎⁎	−7.28 (1.13)⁎⁎⁎	−4.06 (1.24)⁎⁎⁎	−14.79 (2.96)⁎⁎⁎	−6.38 (1.71)⁎⁎⁎
T1 vs T4, Estimate (SE)	0.3 (0.04)⁎⁎⁎	0.4 (0.08)⁎⁎⁎	0.61 (0.09)⁎⁎⁎	−1.87 (1.75)	7.5 (1.24)⁎⁎⁎	1.56 (1.62)	10.97 (3.72)⁎⁎	−2.81 (2.29)
T2 vs T4, Estimate (SE)	0.18 (0.04)⁎⁎⁎	0.07 (0.07)	0.14 (0.08)	0.19 (1.57)	1.2 (1.22)	0.09 (1.45)	1.46 (3.43)	−0.73 (1.99)
T3 vs T4, Estimate (SE)	0.05 (0.03)	0 (0.06)	0.01 (0.08)	−0.16 (1.19)	1.47 (0.97)	0.18 (1.21)	1.45 (2.76)	0.15 (1.61)
Group*T1 vs T4, Estimate (SE)	−0.33 (0.04)⁎⁎⁎	−0.54 (0.09)⁎⁎⁎	−0.64 (0.09)⁎⁎⁎	1.5 (1.63)	6.7 (1.27)⁎⁎⁎	2.24 (1.65)	10.45 (3.74)⁎⁎⁎	−3.05 (2.31)
Group*T2 vs T4, Estimate (SE)	−0.22 (0.04)⁎⁎⁎	−0.25 (0.08)⁎⁎	−0.28 (0.09)⁎⁎	0.4 (1.59)	1.28 (1.39)	0.26 (1.67)	0.63 (3.87)	−0.51 (2.24)
Group*T3 vs T4, Estimate (SE)	−0.06 (0.05)	−0.15 (0.09)	−0.13 (0.1)	0.45 (1.59)	0.99 (1.36)	−0.02 (1.65)	0.57 (3.83)	−0.12 (2.24)
Female vs Male, Estimate (SE)	−0.01 (0.01)	−0.01 (0.03)	0.02 (0.04)	−1.23 (0.66)	−0.45 (0.45)	−1.29 (0.68)	−2.97 (1.51)	0.88 (0.94)
Education years, Estimate (SE)	−0.001 (0.002)	0 (0)	0 (0)	0.02 (0.1)	−0.01 (0.07)	0.14 (0.1)	0.13 (0.23)	0.35 (0.14)⁎
IQ score, Estimate (SE)	0.004 (0.001)⁎⁎	0 (0)	0.01 (0)	−0.02 (0.05)	−0.13 (0.04)⁎⁎⁎	−0.21 (0.05)⁎⁎⁎	−0.36 (0.12)⁎⁎	0.19 (0.08)⁎
Combined medication, Estimate (SE)	−0.03 (0.02)	−0.01 (0.06)	0.04 (0.05)	−0.69 (1.21)	−1.25 (0.67)	−0.08 (1.19)	−2.13 (2.54)	0.65 (1.71)
Haloperidol, Estimate (SE)	−0.01 (0.02)	−0.03 (0.04)	−0.05 (0.05)	2.55 (0.88)⁎⁎	0.65 (0.61)	1.35 (0.87)	4.59 (1.99)⁎	−3.31 (1.25)⁎⁎
Chlorpromazine, Estimate (SE)	−0.01 (0.01)	0.01 (0.03)	0.01 (0.03)	0.82 (0.66)	1.03 (0.49)⁎	1.32 (0.69)	3.37 (1.55)⁎	−1.54 (0.93)
Clozapine, Estimate (SE)	−0.04 (0.02)⁎	−0.03 (0.04)	−0.08 (0.05)	4.42 (0.92)⁎⁎⁎	2.96 (0.64)⁎⁎⁎	5.1 (0.94)⁎⁎⁎	12.61 (2.1)⁎⁎⁎	−6.12 (1.3)⁎⁎⁎
Years of illness, Estimate (SE)	0 (0.03)	0 (0.07)	0 (0.07)	0.13 (1.46)	0.09 (1.1)	0.18 (1.5)	0.4 (3.39)	−0.13 (2.05)
Years of hospitalization, Estimate (SE)	0 (0.03)	0 (0.07)	−0.01 (0.07)	0.13 (1.46)	0.04 (1.1)	0.08 (1.5)	0.25 (3.39)	−0.19 (2.05)
Calls, Estimate (SE)	−0.02 (0.03)	−0.01 (0.07)	−0.04 (0.07)	−0.26 (1.62)	−0.17 (1.15)	−0.15 (1.6)	−0.58 (3.63)	0 (2.22)
Visits, Estimate (SE)	0.06 (0.04)⁎	0.04 (0.09)	0.05 (0.09)	0.08 (1.74)	−0.39 (1.29)	0.12 (1.69)	−0.15 (3.89)	−0.07 (2.34)
Diet, Estimate (SE)	0 (0.03)	0.03 (0.07)	0.04 (0.07)	1.41 (1.52)	0.54 (1.12)	1.59 (1.57)	3.48 (3.51)	−1.34 (2.11)
Residual of random effects, Variance (SE)	0.000 (0.007)	0.000 (0.029)	0.000 (0.005)	0.000 (0.250)	0.000 (0.348)	0.000 (0.132)	0.000 (0.691)	0.000 (0.371)

Note. Random effects of Years of illness, Years of hospitalization, Calls, Visits, Diet were included in the model.

⁎⁎⁎ *p* < .001.

⁎⁎ *p* < .01.

⁎ *p* < .05.

PANSS Negative Symptoms at T4 was lower by 6.7 points (SE = 1.3) compared to T1 and PANSS Total score at T4 was lower by 10.5 points (SE = 3.7) compared to T1.

GAF was not affected by Group Assessment interaction term.

On the other hand, haloperidol consumption was associated with an increase in PANSS Positive and Total symptoms score by 2.6 and 4.59 points respectively and a decreased GAF score by 3.31 points. Clozapine consumption was related to decreased SPS Stimuli scores by 0.04 points, decreased GAF score by 6.12 points, increased Positive, Negative, General and Total symptoms score by 4.42, 2.26, 5.1 and 12.6 points respectively, while Chlorpromazine was associated with increased PANSS Negative and Total symptoms by 1.03 and 3.37 points respectively.

Table 4, Table 5, Table 6 show that the random effects of years of illness, hospitalization, calls, visits, and diet did not significantly affect any test measurements, cognitive domain, symptoms, or functioning, except for social perception (visits were found to be associated with a slight increase of 0.06 points in SPS Stimuli scores; see Table 6).

## 4 Discussion

The IPT has been the first evidence-based rehabilitation program in Greece, conducted in a private psychiatric clinic. In this study, we conducted a randomized controlled trial (RCT) with middle-aged inpatients diagnosed with schizophrenia. Implementation and efficacy evaluation of the full-length IPT program were the main goals. Neurocognition, social perception, psychopathology, and functioning were measured. After the IPT intervention, nearly all domains showed significant improvements. More specifically, statistically significant results from the MCCB's neurocognitive domain (speed of processing, attention/vigilance, neurocognitive composite score, and total composite score), social perception, psychopathology (specifically negative symptoms) and GAF are considered to be important in the possibility of improving cognitive deficits and motivation in individuals with schizophrenia through IPT. Furthermore, the study found that enhancing social perception can improve the everyday functioning of patients. This may strengthen their self-efficacy and community integration (Green and Nuechterlein, 1999).

An additional noteworthy aspect is the comparability between the findings of the current study and those of Rakitzi et al. (2016), as well as the meta-analyses conducted by Roder et al. (2011) and Mueller et al. (2013). Specifically, although being conducted with chronic inpatients, the study found identical results to that of studies with outpatients in terms of neurocognition and social perception (Rakitzi et al., 2016). The available research indicates that IPT exhibits efficacy in enhancing cognitive functioning, namely in the domains of social perception and speed of processing. Furthermore, our research findings, demonstrated superior outcomes compared to the study conducted by Rakitzi et al. (2016) in terms of improving positive, negative and total symptoms as evaluated by the PANSS scale. One plausible explanation is linked to the implementation of all IPT subprograms.

Based on Roder et al.'s (2011) findings, it has been noticed that IPT has the potential to yield more favorable outcomes in terms of neurocognition, social perception, functioning, and negative symptoms when compared to standard care or placebo-attention conditions. According to Roder et al. (2011), individuals undergoing IPT can sustain the average favorable effects for a duration of 8.1 months during follow-up. Moreover, the integration and implementation of all five subprograms of IPΤ has been found to yield improved outcomes in terms of psychopathology and psychosocial functioning. Our study revealed comparable findings in the cohort of chronic middle-aged inpatients.

As mentioned, the current study found similar results to those of Mueller et al. (2013). A typical meta-analysis included 15 controlled IPT studies with 632 schizophrenia patients (Mueller et al., 2013). Mueller et al. (2013) divided research paricipants into two age groups: <40 years and ≥40 years, reporting that IPT improved total cognitive score, neurocognition, and social cognition in middle-aged individuals. Our research yielded comparable results for MCCB's neurocognitive domain and SPS's social perception. This supports the idea that empirically validated IPT can be beneficial to middle-aged patients.

In another study, Aloi et al. (2020) compared IPT to traditional treatment for persistent schizophrenia. Aloi et al. (2020) reported clinical, cognitive, and emotional benefits in the IPT group. These findings indicate that IPT is an effective treatment for individuals with persistent schizophrenia. Furthermore, a recent study conducted by Bossy et al. (2020) implemented IPT inside a hospital setting, in conjunction with additional interventions including Integrated Neurocognitive Therapy (INT) and Cogpack CGP (a computerized neuropsychological cognitive training program). In response to treatment, symptomatology, psychosocial functioning, and neurocognitive performance improved. The findings regarding neurocognition, social perception, and symptomatology among chronic hospital inpatients are similar to those obtained in this study. IPT appears to be useful in treating chronic hospital inpatients.

On the other hand, it is essential to address some shortcomings inherent in this study. The intervention program did not yield significant increases in visual learning, verbal learning, and social cognition as measured by the MCCB. Pathological issues (e.g., cataract; Rogers and Langa, 2010), challenges in establishing learning techniques, and theory of mind impairment (Bora et al., 2009) may make it challenging to achieve positive and significant results.

In addition, it is important to acknowledge that the sample size had been reduced due to dropouts, which could have increased the likelihood of type II errors. Furthermore, TAU's formation might impose certain constraints on managing non-specific factors (such as lack of uniformity and patients' expectations) within the IPT treatment group. Moreover, it is worth noting that the evaluation of social functioning solely through the GAF represents a significant constraint. It is important to recognize its limited ability to detect psychosocial changes and the possibility for misinterpretation with the intensity of symptoms (Mueller et al., 2015; Robertson et al., 2013). Therefore, the inclusion of supplementary indicators of social functioning would have been advantageous. Additionally, it would also be beneficial to examine the domains of social skills and interpersonal problem-solving using specialized assessments in order to evaluate the degree of improvement in these areas following the implementation of IPT. Furthermore, participants' self-report of some factors (e.g., frequency of phone calls) may not fully reflect their impact on the sample measurements, whereas measures of theory of mind (TOM) were not included in this study. Ultimately, the sample comprised predominantly middle-aged male inpatients, and therefore the findings cannot be readily extrapolated to the broader population of individuals with schizophrenia.

Future studies should employ larger sample sizes and evaluate IPT in public hospitals, private psychiatric clinics, and mental health day centers. Moreover, comparing IPT to analogous psychological interventions like Integrated Neurocognitive Therapy (INT; De Mare et al., 2018) will help assess its relative efficacy. Evaluation of its implementation in treatment-resistant schizophrenia (TRS) and non-treatment-resistant schizophrenia (NTRS) would have been enlightening. Rakitzi and Georgila (2019) found that IPT was more effective in Non-Treatment Resistant Schizophrenia (NTRS) than Treatment Resistant Schizophrenia (TRS) in Greek outpatients. However, IPT TRS demonstrated improvements that remained at the 3-month follow-up (Rakitzi and Georgila, 2019).

Furthermore, a thorough analysis of how cardiorespiratory issues, megaloblastic anaemia, diabetes, and cataracts may affect IPT in middle-aged hospitalized individuals will yield significant and informative results. The above issues highlight the necessity for more RCTs among individuals with psychiatric conditions.

Since Mueller et al. (2013) conclude that psychological therapies are essential for middle-aged and older individuals with schizophrenia, IPT can be implemented over all five programs or just the first three (Cognitive Differentiation, Social Perception, and Verbal Communication). Given the persistent advances in neurocognition and social perception, we propose implementing the first three IPT subprograms that address these domains. These three sub-programs are well-organized, time-efficient, and emotionally light, allowing therapeutic social connections without distress. These qualities make them versatile and cost-effective for short or intermediate hospital stays. Multiple IPT sessions can improve cognitive skills, psychopathological symptoms, and functioning (Roder et al., 2011; Rakitzi et al., 2016).

In summary, goal-directed rehabilitation programs, such as IPT, administered by experienced therapists in clinical settings may help middle-aged individuals with schizophrenia recover and reintegrate into society. To establish IPT as a widely used therapy process in clinical psychiatric contexts, its efficacy must be routinely evaluated.

## CRediT authorship contribution statement

**Aikaterini Poulou:** Conceptualization, Data curation, Formal analysis, Funding acquisition, Investigation, Methodology, Project administration, Resources, Validation, Visualization, Writing – original draft, Writing – review & editing. **Fotios Anagnostopoulos:** Conceptualization, Methodology, Project administration, Supervision, Writing – review & editing. **Argiro Vatakis:** Conceptualization, Supervision, Writing – review & editing. **Robert C. Mellon:** Conceptualization, Supervision. **Daniel R. Mueller:** Resources, Supervision.

## Declaration of competing interest

The authors report there are no competing interests to declare.

## Data Availability

The data that support the findings of this study are available on reasonable request from the corresponding author, A.P. The data are not publicly available due to restrictions e.g. they contain information that can compromise research participants' privacy.
